# The effects of sodium alendronate on socket healing after tooth extraction: a systematic review of animal studies

**DOI:** 10.1590/1807-3107bor-2024.vol38.0038

**Published:** 2024-05-13

**Authors:** Nilo Guliberto Martins CHAVARRY, Pedro Villas Boas ABREU, Eduardo Jorge FERES-FILHO, Daniele Masterson Tavares PEREIRA, Lucianne Cople MAIA, Rafael Scaf De MOLON

**Affiliations:** (a) Universidade Federal do Rio de Janeiro – UFRJ, School of Dentistry, Department of Periodontology, Rio de Janeiro, RJ, Brazil; (b) Universidade Federal do Rio de Janeiro – UFRJ, School of Dentistry, Library of Health Science Center, Rio de Janeiro, RJ, Brazil; (c) Universidade Federal do Rio de Janeiro – UFRJ, School of Dentistry, Department of Orthodontic and Pediatric Dentistry, Rio de Janeiro, RJ, Brazil; (d) Universidade Estadual Paulista – Unesp, School of Dentistry at Araçatuba, Department of Diagnosis and Surgery, Araçatuba, SP, Brazil.

**Keywords:** Alendronate, Osteonecrosis, Tooth, Tooth Extraction

## Abstract

The aim of this systematic review was to answer the following question: “Does alendronate, a nitrogen-containing bisphosphonate, improve or impair alveolar socket healing after tooth extraction in animal models”? To this end, a systematic review of the literature was carried out in PubMed, Scopus, LILACS, Web of Science, as well as in the gray literature up to May 2023. Preclinical studies that evaluated alveolar healing after tooth extraction and the intake of sodium alendronate compared with placebo were included. Two investigators were responsible for screening the articles independently, extracting the data, and assessing their quality through the SYRCLE’s RoB tool for randomized trials in animal studies. The study selection process, study characteristics, risk of bias in studies, impact of alendronate on bone healing, and certainty of evidence were described in text and table formats. Methodological differences among the studies were restricted to the synthesis methods. The synthesis of qualitative results followed the Synthesis Without Meta-analysis (SWiM) reporting guideline. From the 19 included studies, five were considered to have low risk, three were of unclear risk, and eleven presented a high risk of bias. The studies were considered heterogeneous regarding alendronate posology, including its dosage and route of administration. Furthermore, a variety of animal species, different age ranges, diverse teeth extracted, and exposure or not to ovariectomy contributed to the lack of parity of the selected studies. Our results indicated that alendronate monotherapy negatively affects the early phase of wound healing after tooth extraction in preclinical studies, suggesting that the bone resorption process after tooth extraction in animals treated with alendronate might impair the bone healing process of the extraction socket. In conclusion, alendronate administration restrains bone resorption, thereby delaying alveolar socket healing . Future studies should be conducted to validate these findings and to better understand the effects of alendronate therapy on oral tissues.

## Introduction

Alendronate has become the drug of choice for osteoporosis treatment because of its recognized anti-remodeling effect in most human skeletal regions, including the hips, spine, femoral neck, tibia, and wrist.^1^ All bisphosphonates (BPs) aim to reduce bone resorption, to improve bone mineral density, and to decrease the risk of fractures.^2^ The main therapeutic target of BPs is the osteoclast, whose inactivation inhibits osteoclastogenesis and prevents osteolysis.^3^ Among oral BPs, alendronate is the most efficient in slowing down skeletal remodeling and turnover. On the other hand, some serious side effects can occur in long-term BP treatment.^4^ It has been shown that alendronate can delay mucosal healing after tooth extraction,^5^ impair alveolar and cortical bone metabolism,^6-8^ compromise implant osseointegration,^9^ and increase the risk of osteonecrosis of the jaw (ONJ).^10-15^

ONJ is the main adverse effect of orally administered BPs.^12,14^ ONJ cases associated with BP therapy have been increasingly reported in dental clinics since 2003.^16^ The incidence of ONJ related to alendronate ranged from 0.01 to 4%.^17^ These pathological cases involve very high morbidity and treatment challenges, thus demanding a major effort in their prevention. It is still not fully understood how suppression of bone remodeling can affect the intrinsic properties of bone metabolism (mineralization, turnover rates, collagen cross-links, microdamage, etc). Although ONJ has been documented to occur spontaneously,^12^ it is most commonly associated with trauma induced by a dental procedure, such as tooth extraction and periodontal/periapical disease.^14,15 18 19^ Research studies have focused on elucidating the role of tooth extraction in triggering the onset of ONJ in patients treated with alendronate for long periods of time.^20^ To date, one cannot predict who is prone to develop ONJ, which represents a dilemma for clinicians dealing with risk assessment. Currently, there are no reliable biochemical markers to guide preventive strategies for ONJ. This condition is characterized by the presence of a non-healing exposed bone in the maxilla or in the mandible, persisting for more than 8 weeks in a patient who has received systemic BP treatment but has not received head and neck radiation therapy.^21,22^ Persistent jaw bone pain, bone enlargement, gingival swelling, jaw bone fractures, pus, and unpleasent mouth odor, are some clinical symptoms described in ONJ.^23^ Despite the side effects, new therapeutic approaches using alendronate have been considered in the treatment of bone resorptive disease in the mouth, especially periodontitis.^24-26^

However, the following questions still need to be properly aswered: Can the antiresorptive effect of alendronate on the alveolar bone prevent crestal bone loss after a tooth extraction? Can we expect to find dead bone in this area? Does alendronate enhance bone fill in socket healing? What is the consequence of decreasing bone remodeling in a dynamic metabolic enviromnent? To deal with these questions, it is important to know the specific characteristics of the jaw bones: The cortical bone of the alveolar region has a high turnover rate of ~25% per year, compared to 1%–2% per year in the tibial or femoral diaphysis.^27^ A very thin mucosal layer covers the bones, which can break easily, leading to exposure in a bacteria-laden environment and a heightened risk of infection. While some authors consider alendronate the therapeutic solution to enhance bone healing and to prevent alveolar bone loss,^24, 28^ other studies posit that alendronate delays the socket healing processes and leads to non-vital bone accumulation.^6, 29^ Additionaly, the final mineralization process and the type of collagen cross-linking in newly formed bones are still not completely understood in the presence of BPs.^30, 31^

Studies using animal models to mimic experimental conditions are widely used in various human-related diseases. The use of experimental tooth extraction and ONJ models permits the study of the molecular mechanisms involved in the immunopathogenesis of diseases and in the healing process that occur after tooth extraction. The tooth extraction model in rabbits and rodents assist in the understanding of events that lead to bone resorption and remodeling resulting from dental extraction. The healing process after tooth extraction in animal models mimic the events that occur in humans and, consequently, are well indicated for the study of bone and soft tissue healing progression. These animal models also contribute to the development of new treatment strategies and supporting decisions about human clinical research. Systematic reviews of preclinical studies are recognized for their importance in identifying interventions with the best preventive or therapeutic potential for testing in randomized clinical studies because they might offer robust and comprehensive descriptions of those animal studies. Therefore, the aim of this systematic review was to answer the following question: “Does alendronate improve or impair alveolar socket healing after tooth extraction”?

## Methodology

### Protocol and registration

This study was conducted at the Systematic Review Facility (SyRF) (https://syrf.org.uk/projects)^32^ as recommended by the Collaborative Approach to Meta Analysis and Review of Animal Experimental Studies (CAMARADES) (https://www.ed.ac.uk/clinical-brain-sciences/research/camarades/about-camarades). This study followed the PRISMA 2020 statement^33^ (https://prisma-statement.org). The study protocol was registered on OSF (Open Science Framework) under the identified number DOI: 10.17605/OSF.IO/FZXTH (https:osf.io). There were no deviations from the initial protocol.

### Eligibility criteria

The controlled vocabulary (MeSH terms) and free keywords in the search strategy (Table 1) were defined based on the elements of the PICOS question: a). Population (P): Experimental laboratory animals (rat, mouse, and rabbit) subjected to tooth extraction; b) Intervention (I): Alendronate therapy; c) Comparison (C): Placebo group; d) Outcome (O): Alveolar healing parameters; e) Study design (S): laboratory animal studies.

**Table 1:** Electronic database and search strategy (2023).

Pubmed	Lilacs and BBO	Web of Science	Scopus	Cochrane
(((Tooth Extraction[mh] OR tooth extraction* [tiab] OR exodontia [tiab] OR dental extraction [tiab] OR Oral surgical procedures [mesh] OR Procedures Maxillofacial [tiab] OR Surgical Procedure Oral [tiab] OR surgery oral [mesh] OR Surgery maxillofacial [tiab] OR oral surgery [tiab] OR Tooth socket [mesh] OR sockets tooth [tiab] OR Alveolar process [mesh] OR Alveolar process [tiab] OR Processes Alveolar [tiab] OR Alveolar Ridge [tiab]))) AND ((Alendronate[mh] OR alendronate[tiab] OR Bisphosphonate* [tiab] OR Diphosphonate[mesh] OR Diphosphonate*[tiab] OR Bisphosphonate Associated Osteonecrosis of the Jaw[mesh])) AND “animal”[Filter])	(tw:(mh:(alendronate or alendronato) or (tw: (alendronate or alendronato) or (tw: (bisphosphonat$ or bisfosfonat$)) or (mh: (difosfonatos)) or (tw: (diphohsphonat$ or difosfonat$)) or (mh: (Bisphosphonate Associated	(“Tooth Extraction*”or exodontia or “dental extraction” or “Oral surgical procedures” or “Procedures Maxillofacial” or “Surgical Procedure Oral” or “surgery oral” or “Surgery maxillofacial” or “oral surgery” or “Tooth socket” or “sockets tooth” or “Alveolar process” or “Processes Alveolar” or “Alveolar Ridge”) and (Alendronate or Bisphosphonate* or Diphosphonate* or “Bisphosphonate Associated Osteonecrosis of the Jaw“)	(“Tooth Extraction*” OR exodontia OR “dental extraction” OR “Oral Surgical procedures” OR “Procedures Maxillofacial” OR “Surgical Procedure Oral” OR “surgery oral” OR “Surgery maxillofacial” OR “oral surgery” OR “Tooth socket” OR “sockets tooth” OR “Alveolar process” OR “Processes Alveolar” OR “Alveolar Ridge”)AND ( alendronate OR bisphosphonate* OR diphosphonate* OR “Bisphosphonate Associated Osteonecrosis of the Jaw” ) ) AND ( TITLE-ABS-KEY ( animal* OR “rabbits” OR “macaca” OR rats OR “mice” OR “dogs” AND (LIMIT-TO (SUBJAREA, “DENT”)) AND (LIMIT-TO (DOCTYPE ,”ar”)	(tooth Extraction [mh] or tooth extraction*[tiab] or exodontia [tiab] or dental extraction [tiab] or Oral Surgery procedures[mesh] or Procedures
	Osteonecrosis of the Jaw)) or (tw:(Bisphosphonate Associated Osteonecrosis of the Jaw or bisfosfonato associado osteonecrose da mandíbula))) AND (tw:(mh:(tooth extraction or extração dentária)) or (tw:(tooth extraction or extração dentária)) or (mh:(exodontia)) or (tw: (exodontia)) or (tw: (dental extraction)) or (mh: (Oral surgical procedures or procedimentos cirúrgicos bucais)) or (tw:(Oral surgical procedures or procedimentos cirúrgicos bucais)) or (tw: (Procedures Maxillofacial) or (tw:(Surgical Procedure Oral)) or (mh:(surgery, oral or cirurgia bucal)) or (tw:(cirurgia bucal)) or (tw:(surgery, oral)) or (tw:(Surgery maxillofacial) or (tw:(oral surgery)) or (mh:(tooth socket or alvéolo dental) or (tw:(tooth socket or alvéolo dental)) or (tw: (sockets tooth)) or (mh: (alveolar process or processo alveolar)) or (tw: (alveolar process or processo alveolar) or (tw: (Processes Alveolar)) or (tw:(alveolar ridge))			Maxillofacial [tiab] or Surgical Procedure
				Oral [tiab] or surgery oral [mesh] or Surgery maxillofacial [tiab] or oral surgery [tiab] or Tooth socket[mesh] or sockets tooth [tiab] or Alveolar process [mesh] or Alveolar process [tiab] or Processes
				Alveolar[tiab] or Alveolar Ridge [tiab]) And (Alendronate [mh] or alendronate[tiab] or Bisphosphonate* [tiab] or Diphosphonate [mesh] or Diphosphonate*[tiab] or Bisphosphonate Associated Osteonecrosis of the Jaw [mesh])

Only experimental animal trials that conducted tooth extraction under the effect of alendronate, in comparison to a placebo, were eligible. No miminum follow-up period was required. Despite the fact that the tested drug aimed to treat osteoporosis, ovariectomy or any other model to simulate postmenopausal phase-induced bone loss was not considered an inclusion criteria, neither was the sex of the animals. The primary outcome of this systematic review was the histopathologic results regarding the expression of alveolar socket healing in terms of quantity and/or quality of soft tissues and bone. In addition, microcomputed tomography was also included. No restrictions on animal species or breed were established. The exclusion criteria were as follows: non-controlled experimental animal trials, editorial letters, pilot studies, historical reviews, and in vitro studies were excluded. Moreover, studies were excluded if the post-extraction alveolar socket was filled with any bone substitute material. Studies that utilized alendronate in combination with other drug and did not have a group of non-mixed drugs were also excluded. Dosage and route of administration were not considered exclusion criteria.

### Information sources and search

Two authors (N.G.M.C. and R.S.M.), guided by a specialized librarian (D.M.T.P.), independently conducted an electronic search up to October 2023 (subsequently updated by alerts) in the PubMed/MEDLINE, Scopus, Web of Science, Latin American and Caribbean Health Sciences Literature database (LILACS), Brazilian Library in Dentistry (BBO), and Cochrane Library (Table 1) to compile the reports for this systematic review. Additional publications were retrieved by manual search of the reference lists from primary studies. There were no restrictions on publication data and languages. The grey literature was utilized to identify eligible studies in the opengrey (opengrey.eu – Grey literature database) and Theses database. Table 1 presents the search strategies, which were appropriately modified for each database.

### Selection process

The retrieved articles were exported to the Rayyan Reference Manager (https://www.rayyan.ai) and duplicates were removed by the program (perfect match) and manually. The selection process was conducted in two phases: In Phase 1, two researchers (N.G.M.C. and R.S.M.) independently examined the titles and abstracts of all retrieved references, applying the inclusion criteria; and in Phase 2, the same two reviewers independently applied the exclusion criteria during the full-text screening. The full texts were evaluated and judged in the entire document. Interrater reliability in the study selection process was determined by Cohen’s kappa, assuming an acceptable threshold value of 0.80.^34^ The disagreement at any stage was resolved by discussion and mutual decision with a third reviewer (LCM).

### Data collection process

Each included article was numbered and catalogued under the name of the first author and year of publication. A customized data extraction form was made to record all information necessary to validate the article in agreement with the eligibility criteria and with the research question. The following topics were used for data extraction: a) details of the study, including year of publication and authors; b) details of the participants, including type, age, and weight of the animals; c) details of the study methods, including study design, experimental groups, ovariectomy, tooth extracted, sample size, and follow-up period; d) details of drug posology, including allometric tests and clinical drug relevance; e) details of socket healing after tooth extraction, including timing of alendronate intervention and healing period; f) details of the outcomes, including histopathologic elements (osteoclast, osteoblast, alveolar bone fill, non-vital bone accumulation, bone remodeling, epithelial coverage, inflammation, blood vessels, collagen apposition, and osteonecrosis) and microcomputed tomography.

### Risk of bias in individual studies

The SYRCLE’s risk of bias tool for animal studies was employed to assess the methodological quality of the selected studies.^35^ Ten entries related to six domains of bias comprised this adapted version of the Cochrane RoB tool: selection bias, performance bias, detection bias, attrition bias, reporting bias, and other biases. All these domains were applied in each study. The Cochrane RoB Tool was the starting point for developing a RoB tool for experimental animal studies. Five entries of Cochrane RoB tool were directy applicable to animal experiments and were adopted (sequence generation, allocation concealment, incomplete outcome data, selective outcome reporting, and other sources of bias). Differences between randomized clinical trials and animal intervention studies were established in order to test whether aspects of animal studies that differed from human randomized controlled trials could cause bias that had not yet been taken into account. Thus, the authors created, adapted, and included the RoB tool in another five entries (baseline characteristics, random housing, blinding in the performance, detection domains, and random outcome assessment).

During data extraction and risk of bias assessment, any disagreement between the reviewers was resolved through discussion, and whenever necessary, by consulting a third reviewer (LCM). The judgment of each entry involved recording ‘yes’ for low risk of bias, ‘no’ for high risk of bias, or ‘unclear’ for either lack of information or uncertainty about the potential for bias, as described in the SYRCLE’s risk of bias tool. Studies were considered to have a ‘low’ risk of bias if there was adequate sequence generation and allocation concealment (key domains). When the study was judged as ‘unclear’ in its key domains, we tried to contact the authors to obtain more information and to allow a definitive judgment about ‘yes’ or ‘no’.

### Synthesis methods and effect measures

The study selection process, study characteristics, risk of bias in studies, impact of alendronate on bone healing, and certainty of evidence were described in text and table formats. Methodological differences among the studies were restricted to the synthesis methods. The synthesis of qualitative results followed the Synthesis Without Meta-analysis (SWiM) reporting guideline.^36^

## Results

### Study selection

**Figure 1:**
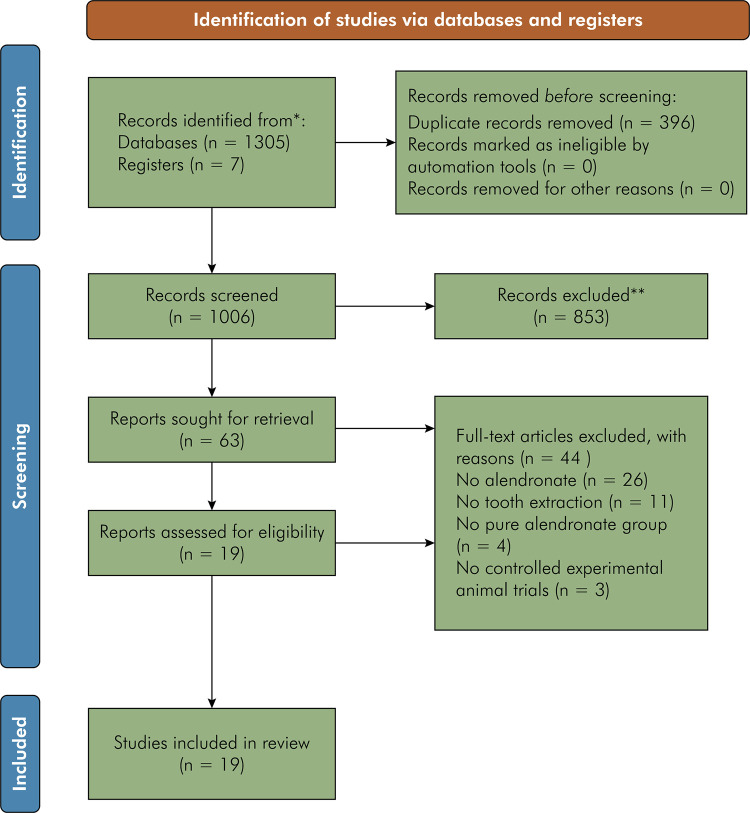
Flowchart of the study according to the PRISMA statement (2020).

After database screening and duplicate removal, 1,305 studies were identified (Figure 1). After reading the titles, 63 articles remained, and after careful abstract assessement, 44 reports were excluded (Table 2) due to the following reasons: reports that did not have a pure alendronane group; and studies that did not perform tooth extraction. The full text of the remaining 19 studies was retrieved^10, 11, 37-53^ and included in this systematic review.

*Consider, if feasible to do so, reporting the number of records identified from each database or register searched (rather than the total number across all databases/registers).

**If automation tools were used, indicate how many records were excluded by a human and how many were excluded by automation tools.

**Table 2:** List of studies excluded from phase 2.

Authors	Title, Journal, year	Reason for exclusion
Allen MR.	The effects of bisphosphonates on jaw bone remodeling, tissue properties, and extraction healing. Odontology. 2011;99(1):8-17.	No pure alendronate
Attar BM, Razavi SM, Daneshmand M, Davoudi A.	Protective effects of resveratrol against osteonecrosis at the extraction site in bisphosphonate-treated rats. International Journal of Oral and Maxillofacial Surgery. 2020;49(11):1518-22.	No pure alendronate
Bi YM, Gao YM, Ehirchiou D, Cao CZ, Kikuiri T, Le A, et al.	Bisphosphonates Cause Osteonecrosis of the Jaw-Like Disease in Mice. American Journal of Pathology. 2010;177(1):280-90	No alendronate
Dayisoylu EH, Şenel F, Üngör C, Tosun E, Çankaya M, Ersöz S, et al.	The effects of adjunctive parathyroid hormone injection on bisphosphonate-related osteonecrosis of the jaws: an animal study. Int J Oral Maxillofac Surg. 2013;42(11):1475-80.	No alendronate
Demircan S, İşler SC.	Histopathological Examination of the Effects of Local and Systemic Bisphosphonate Usage in Bone Graft Applications on Bone Healing. Journal of Maxillofacial and Oral Surgery. 2021;20(1):144-8.	No tooth extraction
Develi T, Uckan S, Bayram B, Deniz K, Erdem SR, Ozdemir BH, et al.	Preventive and therapeutic effects of relaxin on bisphosphonate-related osteonecrosis of the jaw: An experimental study in rats. Brazilian Dental Science. 2020;23(1).	No tooth extraction
Duygu G, Yalcin-Ülker GM, Günbatan M, Soluk-Tekkesin M, Özcakir-Tomruk C.	Evaluation of Preventive Role of Systemically Applied Erythropoietin after Tooth Extraction in a Bisphosphonate-Induced MRONJ Model. Medicina (Kaunas). 2023;59(6).	No alendronate
Fouda N, Caracatsanis M, Kut IA, Hammarström L.	Mineralization disturbances of the developing rat molar induced by mono- and bisphosphonates. J Biol Buccale. 1991;19(1):106-15.	No alendronate
Frizzera F, Verzola MHA, Molon RS, Oliveira GJPL, Giro G, Spolidorio LC, et al.	Evaluation of bone turnover after bisphosphonate withdrawal and its influence on implant osseointegration: an in vivo study in rats. Clinical Oral Investigations. 2019;23(4):1733-44.	No tooth extraction
Hidaka K, Mikuni-Takagaki Y, Wada-Takahashi S, Saita M, Kawamata R, Sato T, et al.	Low-Intensity Pulsed Ultrasound Prevents Development of Bisphosphonate-Related Osteonecrosis of the Jaw-Like Pathophysiology in a Rat Model. Ultrasound Med Biol. 2019;45(7):1721-32.	No pure alendronate
Hokugo A, Kanayama K, Sun S, Morinaga K, Sun Y, Wu Q, et al.	Rescue bisphosphonate treatment of alveolar bone improves extraction socket healing and reduces osteonecrosis in zoledronate-treated mice. Bone. 2019;123:115-28.	No alendronate
Hokugo A, Sun S, Park S, McKenna CE, Nishimura I.	Equilibrium-dependent bisphosphonate interaction with crystalline bone mineral explains anti-resorptive pharmacokinetics and prevalence of osteonecrosis of the jaw in rats. Bone. 2013;53(1):59-68.	No alendronate
Huang J, Wang L, Tian W.	Small Extracellular Vesicles Derived from Adipose Tissue Prevent Bisphosphonate-Related Osteonecrosis of the Jaw by Promoting Angiogenesis. Int J Nanomedicine. 2021;16:3161-72.	No alendronate
Imada M, Yagyuu T, Ueyama Y, Maeda M, Yamamoto K, Kurokawa S, et al.	Prevention of tooth extraction-triggered bisphosphonate-related osteonecrosis of the jaws with basic fibroblast growth factor: An experimental study in rats. PLoS One. 2019;14(2):e0211928.	No alendronate
Jabbour Z, El-Hakim M, Henderson JE, de Albuquerque RF, Jr.	Bisphosphonates inhibit bone remodeling in the jaw bones of rats and delay healing following tooth extractions. Oral Oncol. 2014;50(5):485-90.	No alendronate
Jeffcoat M.	Safety of oral bisphosphonates: controlled studies on alveolar bone. International journal of oral & maxillofacial implants [Internet]. 2006; 21(3):[349-53	Non-controlled experimental animal trials
Kaynak D, Meffert R, Bostanci H, Günhan O, Ozkaya OG.	A histopathological investigation on the effect of systemic administration of the bisphosphonate alendronate on resorptive phase following mucoperiosteal flap surgery in the rat mandible. J Periodontol. 2003;74(9):1348-54.	No tooth extraction
Kim JW, Cha IH, Kim SJ, Kim MR.	Biomarkers for Bisphosphonate-Related Osteonecrosis of the Jaw. Clinical Implant Dentistry and Related Research. 2016;18(2):281-91.	No alendronate
Kobayashi M.	Inhibitory effects of bisphosphonates on the resorption of alveolar bone in rats]. Nihon Shishubyo Gakkai Kaishi. 1985;27(1):51-64.	No tooth extraction
Koneski F, Popovic-Monevska D, Gjorgoski I, Krajoska J, Popovska M, Muratovska I, et al.	In vivo effects of geranylgeraniol on the development of bisphosphonate-related osteonecrosis of the jaws. J Craniomaxillofac Surg. 2018;46(2):230-6.	No alendronate
Kun-Darbois JD, Libouban H, Mabilleau G, Pascaretti-Grizon F, Chappard D.	Bone mineralization and vascularization in bisphosphonate-related osteonecrosis of the jaw: an experimental study in the rat. Clinical Oral Investigations. 2018;22(9):2997-3006.	No alendronate
Kuroshima S, Nakajima K, Sasaki M, I T, Sumita Y, Asahara T, et al.	Systemic administration of quality- and quantity-controlled PBMNCs reduces bisphosphonate-related osteonecrosis of jaw-like lesions in mice. Stem Cell Res Ther. 2019;10(1):209.	No alendronate
Lazar AC, Ilea A, Onisor F, Bel L, Sarpatoczi O, Purdoiu R, et al.	Effects of Bisphosphonates on the Jaw Bone in Dental Extractions Histological and biochemical aspects in an animal model. Revista De Chimie. 2016;67(4):692-5.	No alendronate
Mada EY, Santos AC, Fonseca AC, Biguetti CC, Neves FT, Saraiva PP, et al.	Effects of green tea and bisphosphonate association on dental socket repair of rats. Arch Oral Biol. 2017;75:1-7.	No alendronate
Marino KL, Zakhary I, Abdelsayed RA, Carter JA, O’Neill JC, Khashaba RM, et al.	Development of a rat model of bisphosphonate-related osteonecrosis of the jaw (BRONJ). J Oral Implantol. 2012;38 Spec No:511-8.	No alendronate
Migliorati CA, Saunders D, Conlon MS, Ingstad HK, Vaagen P, Palazzolo MJ, et al.	Assessing the association between bisphosphonate exposure and delayed mucosal healing after tooth extraction. Journal of the American Dental Association. 2013;144(4):406-14	Non-controlled experimental animal trials
Mitsimponas KT, Moest T, Iliopoulos C, Rueger T, Mueller C, Lutz R, et al.	Search for a reliable model for bisphosphonate-related osteonecrosis of the jaw: establishment of a model in pigs and description of its histomorphometric characteristics. British Journal of Oral and Maxillofacial Surgery. 2016;54(8):883-8	No alendronate
Moraes MB, Lopes GdS, Nascimento RD, Gonçalves FdCP, Santos LMd, Raldi FV.	Use of ozone therapy together to low power laser in osteonecrosis induced bisphosphonates: clinical case. Braz dent sci. 2016;19(1):129-34	Non-controlled experimental animal trials
Oliveira CCD, Barros Silva PGD, Ferreira AEC, Gonçalves RP, Sousa FBD, Mota MRL, et al.	Effects of dexamethasone and nimesulide on bisphosphonate-related osteonecrosis of the jaw: An experimental study. Archives of Oral Biology. 2017;83:317-26.	No alendronate
Özalp Ö, Toru HS, Altay MA, Sindel A.	Evaluation of the Efficacy of EDTA Chelation on Alveolar Bone Healing After Ultrasonic and Conventional Surgery Under Bisphosphonate Medication: A Rat Model. Journal of Oral and Maxillofacial Surgery. 2019;77(10):1982-9	No alendronate
Pan QT, Zang XL, Sun ZW, Pan MQ, Zhu XM, Li ZY.	The role of semaphorin 4D in the mechanism of bisphosphonate-related osteonecrosis of the jaw in rats]. Shanghai Kou Qiang Yi Xue. 2022;31(6):625-31	No alendronate
Pautke C, Kreutzer K, Weitz J, Knödler M, Münzel D, Wexel G, et al.	Bisphosphonate related osteonecrosis of the jaw: A minipig large animal model. Bone. 2012;51(3):592-9.	No alendronate
Pazouki MR, Golestaneh A, Aminzadeh A	Effectiveness of local application of simvastatin for prevention of bisphosphonate-related osteonecrosis of the jaw: An animal study on rats. Journal of Oral and Maxillofacial Surgery, Medicine, and Pathology. 2022	No alendronate
Preidl RHM, Amann K, Weber M, Schiller M, Ringler M, Ries J, et al	Lineage-associated connexin 43 expression in bisphosphonate-exposed rat bones. Journal of Cranio-Maxillofacial Surgery. 2021;49(8):738-47	No tooth extraction
Santamaria Júnior M, Fracalossi ACC, Consolaro MFMO, Consolaro A	Influence of bisphosphonates on alveolar bone density: a histomorphometric analysis. Braz oral res. 2010;24(3):309-15.	No tooth extraction
Song Z, Dong W, Yin L, Liu J, Sun H, Qi M	Effect of thalidomide on development of bisphosphonate-related osteonecrosis of the jaws in rats]. Nan Fang Yi Ke Da Xue Xue Bao. 2015;35(8):1084-9	No alendronate
Taniguchi N, Osaki M, Onuma K, Ishikawa M, Ryoke K, Kodani I, et al	Bisphosphonate-induced reactive oxygen species inhibit proliferation and migration of oral fibroblast: A pathogenesis of bisphosphonate-related osteonecrosis of the jaw. Journal of Periodontology. 2020;91(7):947-55	No alendronate
Tseng HC, Kanayama K, Kaur K, Park SH, Park S, Kozlowska A, et al	Bisphosphonate-induced differential modulation of immune cell function in gingiva and bone marrow in vivo: role in osteoclast-mediated NK cell activation. Oncotarget. 2015;6(24):20002-25	No alendronate pure
Wesselink PR, Beertsen W	Ankylosis of the mouse molar after systemic administration of 1-hydroxyethylidene-1,1-bisphosphonate (HEBP). J Clin Periodontol. 1994;21(7):465-71	No tooth extraction
Yaffe A, Herman A, Bahar H, Binderman I	Combined local application of tetracycline and bisphosphonate reduces alveolar bone resorption in rats. J Periodontol. 2003;74(7):1038-42	No tooth extraction
Yaffe A, Iztkovich M, Earon Y, Alt I, Lilov R, Binderman I	Local delivery of an amino bisphosphonate prevents the resorptive phase of alveolar bone following mucoperiosteal flap surgery in rats. J Periodontol. 1997;68(9):884-9	No tooth extraction
Yifat M, Hila E, Avraham H, Inchingolo F, Mortellaro C, Peleg O, et al	Histologic and Radiographic Characteristics of Bone Filler Under Bisphosphonates. J Craniofac Surg. 2019;30(4):1085-8	No tooth extraction
Zang X, He L, Zhao L, He Y, Xiao E, Zhang Y	Adipose-derived stem cells prevent the onset of bisphosphonate-related osteonecrosis of the jaw through transforming growth factor β-1-mediated gingival wound healing. Stem Cell Res Ther. 2019;10(1):169	No alendronate
Zhang WY, Xuan B, Guo YX, Zhang J	Changes of distal-less homeobox genes 5 and Msh homeobox 1 in a rat model of bisphosphonate related osteonecrosis of the jaw]. Zhonghua Kou Qiang Yi Xue Za Zhi. 2018;53(7):466-9	No alendronate

### Risk of bias within studies

**Figure 2:**
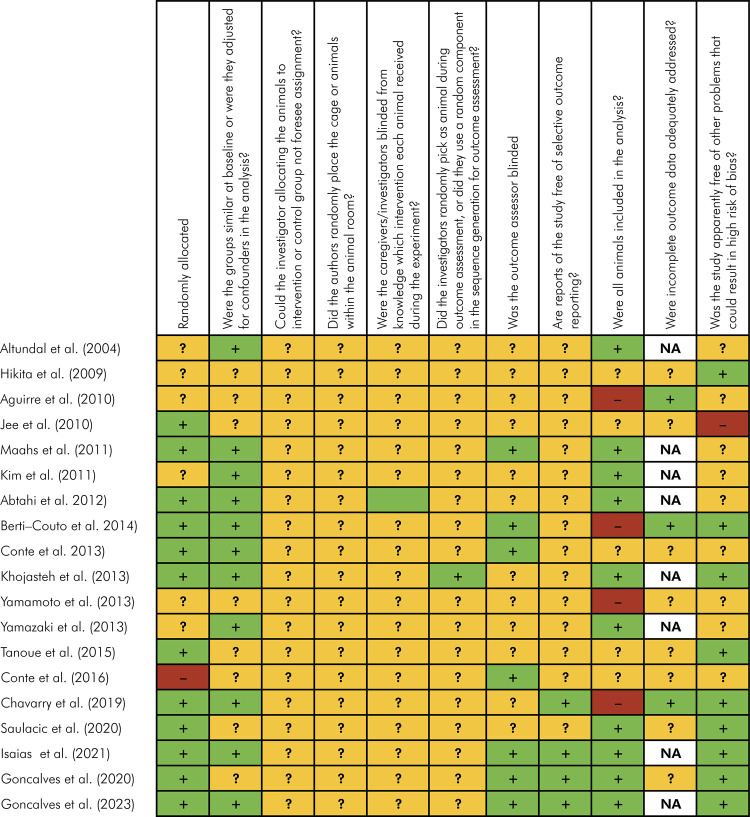
Risk of bias of individual animal studies included. Items scored as low risk (green /+), high risk (red/-), or unclear (yellow/?) risk of bias.

The risk of bias assessment of the selected studies is presented in Figure 2. In summary, from the 19 eligible studies, five^39,42,45,52-53^ were considered to have “low’ risk of bias in the SYRCLE’s RoB tool, three studies^40,43,49^ were considered to have “unclear” risk of bias, and 11 studies were considered to have “high” risk of bias.^10,11,37,38,41,44,46-48,50,51^ Twelve studies reported the randomization process for group allocation, but the sequence generation and the allocation concealment were not described in any of them.^11,37,39,40,42,44,45,47-49,52-53^ In five^39,42,45,52-53^ studies, the authors described the characteristics of the animals in sufficient detail to consider the experimental groups to be similar at baseline and to judge the study as having “low”’ risk of bias. Eleven articles did not report any processes to create and conceal allocation sequence, so they were considered to be unclear and to have a “high” risk in the selection bias domain.^10,11,37,38,41,44,46-48,50,51^ The authors did not provide information about the random placement of cages or animals within the animal room/facility. So, all of them received an unclear score in this domain. Only two studies^37,42^ reported that the investigator was blinded to the treatment allocation, and five studies stated that the outcome evaluator was blinded.^11,37,39,41,47^ The incomplete outcome data were adequately addressed in only three studies.^10,39,50^ All reports were unclear about selective outcome reporting, and four studies^39,43,45,49^ were apparently free of other problems that could result in high risk of bias.

### Characteristics of included articles and synthesis of the ressults

The characteristics of the 19 selected studies^10,11,37-53^ are listed in Tables 3A and 3B. A total of 798 animals had been included in all eligible articles, distributed as 157 Spraguey-Dawley rats, 259 Wistar rats, 176 Holtzmann rats, 141 C57BL/6 mice, 12 mongrel dogs, 15 beagle dogs, and 38 New Zealand rabbits. The rat was the animal chosen in 15 out of 17 reports. In 12 studies,^10,11,37,38,42-44,46,49-51,53^ the age of the animals at baseline ranged from 4 to 10 weeks, and in five studies,^39,40,45,47,48^ at the end of the experiments, the animals were 12 weeks old or older. Two studies did not provide the age of the animals^41,52^ and four studies^10,44,49,50^ did not give the mean weight of the animals. The maxillary molars were extracted in eight reports,^37,39,43,44,46,47,49,51^ the mandibular molars were extracted in eight reports,^10,11,38,41,42,45,48,50^ and the mandibular and maxillary premolars were extracted in one study.^40^ Ovariectomy was performed in three studies.^40,44,51^ The clinically equivalent dose of the alendronate used in the animal studies were assessed by an allometric test, using a metabolic dose.^54^ There was a great variability in the doses given to the animals, when compared to human doses. One study^47^ prescribed a dose 333-fold under the clinically equivalent dose, while another study^51^ prescribed a dose 253-fold above it. Only four studies^39,40,45,52^ were comparable to the 70 mg/week oral dose in postmenopausal women, and nine studies^11,37,38,42,44,46,50,51,53^ exceeded the principle of the tenfold safety factor, proposed by Freed (2006).^55^ The subcutaneous route of administration was used in 12 studies,^10,11,37-39,41,43,44,46,49-51^ oral gavage was used in six studies,^40,42,45,47,52,53^ and one study used local administration.^48^ The duration of alendronate therapy ranged from 5 days^43^ to one year^45^ and the socket healing period ranged from 3^11,43,46,49^ to 105 days.^47^

**Table 3:** Summary of the selected studies in this systematic review.

Authors, year, reference	Animal model	Sample size (number)	Animal age at baseline and at the end of the study	Weight of the animals	Groups	Tooth extracted	Ovariectomy (OVX)	Dosage and route of administration	Allometric test	Dose applied (mg/week)	Was the dose applied clinically relevant?	Dose applied X human dose	Alendronate administration period (before and/or after extraction)	Socket healing period
Altundal H et al., 2004^38^	Male Wistar rats	60	Baseline: 7 and 8 weeks; End: 10/12 weeks	0.204 kg	3 groups: Control, saline and alendronate (ALD)	First right mandibular molar	No	0,25 mg/kg/day/	no	0.35	no	12 times more	2 and 4 weeks after tooth extraction	2 and 4 weeks
Hikita H et al., 2009^43^	Male Wistar rats	60	Baseline: 5 weeks; End: 7 weeks	0.110-0.120 kg	2 groups: Control and ALD	Second right maxillary molar	No	1 mg/kg/every 4 days/Subperiosteal	no	0.24	no	8 times more	2 days before and 3,7,10, 14 days after	3,7,10 and 14 days
Aguirre JI et al., 2010^10^	Male Sprague-Dawley rats	73	Baseline: 9 weeks; End: 15/16/21/30 weeks	Not informed	3 groups: Control, ALD 15 µg and ALD 150 µg	First left mandibular molar	No	0.15 mg/kg/twice week/SC	body mass	0.105	yes	2 times more	3-4 weeks before and 10, 21, 35, 70 days after	10, 21, 35 and 70 days
Jee JH et al., 2010^44^	Female Sprague-Dawley rats	20	Baseline: 5 weeks; End: 7/9/11 weeks	Not informed	3 groups: Sham, OVX-saline; OVX + ALD	First left maxillary molar	Yes	1.0 mg/kg/day/SC	no	1.05	no	32 times more	2, 4 and 6 weeks after	2, 4 and 6 weeks
Maahs MP et al., 2011^47^	Female Wistar rats	31	Baseline: 20 weeks; End: 57 weeks	0.240 kg	3 groups: Control, ALD and zolendronate	Maxillary right molars	No	0.05 mg/kg/week/ oral gavage	no	0.012	no	333 times less	45 days before and 105 days after	105 days
Kim JH et al., 2011^46^	Male Sprague-Dawley rats	24	Baseline: 4 weeks; End: 8/9/10/12 weeks	0.130-0.140 kg	2 groups: Control and ALD	First maxillary molars bilaterally	No	5.0 mg/kg/3 times week/SC	no	2.1	no	72 times more	4 weeks before and 3, 7, 14, 28 days after	3, 7, 14 and 28 days
Abtahi J et al., 2012^37^	Male Sprague-Dawley rats	40	Baseline: 10 weeks; End: 14 weeks	0.383-0.440 kg	4 groups: Control, ALD, ALD + dexamethasone and dexamethasone	First left maxillary molar	No	0.2 mg/kg/day/SC	no	0.616	no	11 times more	14 days after	14 days
Berti–Couto SA et al., 2014^39^	Female Holtzmann rats	44	Baseline: 20 weeks; End: 48 weeks	0.250 kg	4 groups: Control, ALD, ALD + corticosteroid, ALD + diabetes induced	Maxillary right molars	No	0.05 mg/kg/week/SC	no	0.125	yes	equivalent	90 days before and 21 days after	21 days
Conte Neto N et al., 2013^11^	Male Holtzmann rats	20	Baseline: 8 weeks; End: 23/30 weeks	0.200 kg	2 groups: Control and alendronate	First left mandibular molar	No	1.0 mg/kg/day/ SC	no	1.4	no	37 times more	60 days before and 3 or 28 days after	3 and 28 days
Khojasteh A et al., 2013^45^	Mongrel dogs	12	Baseline: 1-2 years; End: 2/4 months	17-24 kg	3 groups: Control, ALD, pamidronate	First and second right mandibular premolar	No	3.5 mg/kg/week/oral	no	73.5	yes	equivalent	01 year before extractions	2 months
Yamamoto-Silva FP et al., 2013^50^	Male Wistar rats	30	Baseline: 7 weeks; End: 10/11/12 weeks	Not informed	2 groups: Control and ALD	Second mandibular molar	No	2.5 mg/kg/day/SC	no	3.5	no	93 times more	14 days before and 7, 14, 21 after	7, 14 and 21 days
Yamazaki T et al., 2013^51^	Male Wistar rats	30	Baseline: 6 weeks; End: 7/9/10 weeks	0.180 kg	2 groups: OVX-control and OVX-ALD	Second maxillary molars	Yes	200 mg/kg/once/ intraperitoneal	no	9	no	253 times more	One injection one week before	1, 2 and 4 weeks
Tanoue R et al., 2015^49^	C57BL6/J male mice	105+36	Baseline: 8 weeks; End: 8/10/11 weeks	Not informed	15 groups: ALD, parathormone and placebo	First right maxillary molar	No	0.1 mg/kg/day/SC	no	0.16	no	3 times less	3,5,7,10, 21 days after and 5, 10, 21 days after	3,5,7,10, 21 days and 5, 10, 21 days
Conte Neto N et al., 2016^41^	Male Holtzmann rats	12	Not informed	0.200 kg	2 groups: Control and ALD	First left mandibular molar	No	1 mg/kg/week/SC	no	0.2	no	5 times more	60 days before and 28 days after extraction	28 days
Chavarry NGM et al., 2019^40^	New Zealand white rabbits	38	Baseline: 6 months; End: 4/10 weeks	4 kg	3 groups: Sham, OVX and ALD+OVX	Maxillary and mandibular first premolars bilaterally	Yes	16 mg/kg/week/oral gavage	body mass	2.03	yes	Equivalent	4 and 10 weeks before extraction	4 and 10 weeks
Saulacic N et al., 2020^48^	Male beagle dogs	15	Baseline: 12 months; End: 1/2/8 weeks	Not informed	4 groups: Control, and ALD at 3 different dosages 0.5, 1 and 2 mg/mL	Mesial root of mandibular premolar	No	0.5, 1 and 2 mg/ml/topical application	no	0.2	no	5 times more	1, 2 and 8 weeks after extraction	1, 2 and 8 weeks
Isaias PHC et al.^52^	Male Wistar rats	48	Not informed	0.180-0.200 kg	8 groups: discontinuous ALN (2.5, 5.0, and 7.5 mg/kg), continuous ALN (2.5, 5.0, and 7.5 mg/kg), positive control (ZA; 0.2 mg/kg), and negative control (saline; 0.1 mL/kg)	Lower left first molar	No	ALN (2.5, 5.0, and 7.5 mg/kg), positive control (ZA; 0.2 mg/kg), and negative control (saline; 0.1 mL/kg) Oral gavage	Yes	2.5, 5.0, and 7.5	Yes	Equivalent	ALN solution, by oral gavage at days 0, 7, 14, 21, 28, 35, 42, 49, 56, and 63.	4 weeks
Goncalves FC et al., 2022^42^	Male Holtzman rats	60	Baseline: 10 weeks;	0.190-0.210 kg	6 groups: Control, ALD, ALD + saline, ALD + strontium ranelate; ALD + saline (60 days) and ALD + strontium (60 days)	First mandibular molar bilaterally	No	1 mg/kg/day/oral gavage	no	1.4	no	37 times more	60 days before extraction and 30 days after	30 days
			End: 23/30 weeks											
Goncalves FC et al., 2023^53^	Male Holtzman rats	40	Baseline: 10 weeks;	0.280-0.300kg	4 groups: -Control Group (saline solution); -Alendronate Group (ALN);	First mandibular molar	No	1 mg/kg/day/oral gavage	no	1.4	no	37 times more	60 days before extraction and 30 days after	30 days
			End: 18 weeks		-Alendronate/ Red Laser Group (ALN/RL); irradiation with GaAlAs laser -Alendronate/ Infrared Laser Group (ALN/IRL): irradiation with a GaAlAs laser									

Histologic analyses were perfomed in all studies, except in two^40,44^ (Tables 4A and 4B). In 11 out of 12 studies that measured bone fill, alendronate was associated with an early-stage delay in the healing process.^10,11,38,41-43,48,50-53^ For instance, a study conducted by Aguirre et al.^10^ showed that moderate dosage of alendronate decreased 55% of woven bone volume compared to the control group, and reduced 75% of woven bone volume at high dosages. Alendronate also reduced the eroded surface of the interalveolar septum by 90%. Another study performed by Conte Neto et al.^11^ showed that animals treated with alendronate retained the interradicular septum, which was associated with bone necrosis and infection. Altundal et al.^38^ showed that alendronate decreased bone fill and resulted in thicker buccal and lingual alveolar bone in the alveolar socket. Yamamoto-Silva et al.^50^ stated that alendronate decreases bone formation at day 7 (0%), 14 (≈ 10%), and 21 (≈ 20%). A small amount of new bone formation was observed compared to the control group at days 7 (≈ 40%), 14 (≈ 60%), and 21 (≈ 60%). On the other hand, Aguirre et al.^10^ demonstrated that moderate and high doses of alendronate increased cancellous and cortical bone mass (35.94 ± 10.71 and 36.01 ± 10.08) compared to the control group (19.61 ± 4.32), presumably due to their inhibitory effect on bone resorption. Yamazaki et al.^51^ showed that alendronate-treated rats exhibited a small amount of new immature bone in the extraction socket (≈25%) compared to the control group (≈ 50%) at day 7. After 14 days, newly formed bone in the socket had an irregular mesh and granulation tissue. The newly formed bone (≈ 60%) lacks the bone-like fiber bundle compared to control animals (≈ 80%). Finally, at day 28, alendronate decreased bone formation (≈ 70%) compared to the control (≈ 100%).

**Table 4:** Summary of the selected histologic assessment.

Authors, year, reference	Osteoclast	Osteoblast	Woven bone / osteoid / Granulation tissue	Socket bone fill / bone healing	Remodeling Interalveolar septum / Alveolar bone	Empty osteocyte lacunae/bone sequestrum	Blood vessel	Collagen apposition/connective tissue	Epithelial coverage	Inflammation	Infection/ osteonecrosis
Altundal H et al., 2004^38^	Alendronate significantly decreased the number of osteoclasts. An average of 2.6 ± 1.8 osteoclasts per measured area appeared in the alveolar crest region in the control group compared with an average of 1.3 ± 0.7 osteoclasts	Day 14 and 28: Alendronate decreased the number of osteoblasts bordering alveolar bone and osteoblast activity. Osteoid formation both at days 14 and 28 was greater in the control group than in the alendronate group.	Days 14 and 28: Alendronate decreased osteoid surface	Days 14 and 28: Alendronate decreased bone fill and resulted in thicker alveolar bone in the buccal and lingual region of the extraction socket	Days 14 and 28: Alendronate decreased interalveolar septum, number of osteoclasts, and resorptive lacunae, and increased alveolar bone thickness by preventing buccal and lingual bone loss by 95% and 98%, respectively	n/a	n/a	n/a	n/a	n/a	n/a
Hikita H et al., 2009^43^	Days 3 and 7: Alendronate decreased the number of osteoclasts per bone surface and tissue area; Day 14: Alendronate increased the number and enlarged; osteoclasts tended to be more deformed and larger, and their nuclei tended to be greater in number and aggregated to a greater extent in the alendronate group.	na	Alendronate decreased woven bone and increased granulation tissue. At 7 days, the extraction socket was completely filled with granulation tissue, and almost no new bone had formed in the extraction socket in the BP group. Ten and 14 days after tooth extraction the extraction socket was filled with newly formed bone.	Day 7: Alendronate decreased bone fill at day 7 but at 14 days more prominent resorption was observed in the control group compared to the alendronate group with clearly higher new bone density in the alendronate group.	Days 7,10, and 14: Alendronate decreased interalveolar septum resorption; Day 14: Alendronate decreased alveolar bone resorption. The proportion of newly formed bone increased in the alendronate group, ranging from 16.90 ± 8.66 at 3 days after extraction; 35.23 ± 9.62 (7 days); 64.76 ± 13.00 (10 days); and 74.99 ± 4.02 (14 days)	n/a	n/a	n/a	n/a	n/a	n/a
Aguirre JI et al., 2010^10^	Days 10 and 70: At 10 days, no differences were observed on eroded and osteoclast surfaces between the control group and the groups treated with either dose of alendronate.	Day 10: Alendronate showed strong tendency to decrease osteoblast surface and number;	Day 10: Moderate dosage of alendronate decreased 55% of woven bone volume compared to the control group and 75% at higher doses; Alendronate significantly decreased the osteoid surface, decreased mineralizing surface (45%), and bone formation rate (90%)	Day 70: Both doses of alendronate increased cancellous and cortical bone mass (35.94 ± 10.71 and 36.01 ± 10.08) compared to the control group (19.61 ± 4.32) presumably due to their inhibitory effect on bone.	Day 10: Alendronate decreased the eroded surface in the interalveolar septum (90%); Alendronate had higher interalveolar septum volume	Day 10: ROI 2: Alendronate displayed a 2.5-fold increase in the percentage of empty osteocyte lacunae	Day 10: Alendronate decreased blood vessel area (60%), number (45%) and perimeter (40%) The decline in these parameters was approximately 30% compared to vehicle-treated rats	Day 10: In 3 out of 9 alendronate-treated rats the interdental alveolar bone exceeded the superficial surface of the socket, which was occupied by inflammatory tissue and not covered by oral epithelium. In these cases, the interdental bone appeared to be exteriorized in the oral cavity.	n/a	n/a	
	Alendronate decreases eroded surface	ALN treatment induced a significant decrease in osteoblast surface (50%), mineralizing surface (45%), bone formation rate (90%) and eroded surface (90%)			At day 70, the socket was almost completely filled with mature lamellar bone in alendronate group.						
Maahs MP et al., 2011^47^	n/a	n/a	n/a	n/a	n/a	n/a	Day 105: Vascular endothelial growth factor (VEGF) was not statistically significant compared to the control group	Day 105: Connective tissue (37.68 ± 9.36) was not statistically significant compared to the control group (36.70 ± 7.67)	Day 105: Alendronate increased loss of mucosal integrity by 72.7% (7 rats out of 10)	Day 105: Inflammatory infiltrate was present in 7 animals out of 11 (63.6%) without statistically significant differences.	Day 105: Alendronate did not induce bone osteonecrosis in any of the animals.
Kim JH et al., 2011^46^	Day 3,7,14 and 28: The number of osteoclasts did not differ from the control group, but there is reduction of osteoclast function in the alendronate group.	Day 7: Osteoblast marker Collagen 1 was non-significant between alendronate and control groups. The function of osteoblast was not delayed	Day 3,7 14 and 28: Woven bone is non-significant	Day 3,7 14 and 28: There is no difference between Alendronate and control group in new bone formation in any of the periods evaluated.	Day 7: Alendronate suppresses inter alveolar septum bone remodeling	Day 7: Alendronate significantly increases empty osteocytic lacunae (74.33 ± 10.50) compared to control (41.67 ± 15.50)	n/a	Day 7: Connective tissue was not statistically significant	n/a	n/a	n/a
Abtahi J et al., 2012^37^	n/a	n/a	n/a	n/a	n/a	Day 14: Alendronate group showed small bone sequestra (3 out of 10 rats).	n/a	n/a	Day 14: The alendronate and control groups showed an intact overlying mucosa without differences between them.	Day 14: Alendronate decreased inflammation	Day 14: Alendronate did not induce osteonecrosis
Berti–Couto SA et al., 2014^39^	n/a	n/a	n/a	n/a	n/a	Empty osteocytic lacunae were not statistically significant when comparing alendronate and control groups.	n/a	Connective tissue in the alendronate group (38.6%) was not statistically significant compared to the control group (29.36%)	Total mucosal coverage was not significant between the alendronate (12.12%) and control groups (13.37%)	Inflammatory infiltrate was non-significant when comparing the alendronate (2.53%) and control groups (0.95%)	Alendronate did not induce osteonecrosis
Conte Neto N et al., 2013^11^	n/a	n/a	Day 3 and 28: The amount of connective tissue was non-significant when comparing alendronate and control animals	Day 28: Alendronate exhibited only slight bone formation, restricted to the apical socket area	Day 3: New bone present in both groups. Day 28: Animals treated with alendronate demonstrated retention of the interadicular septum, which was associated with bone necrosis and infection.	n/a	Day 3: The number of blood vessels was not significant compared to the control group. Day 28:Alendronate decreased the number of blood vessels	Day 28: Alendronate showed partial epithelial coverage exhibiting areas of bone exposure. Control animals showed total epithelial coverage without signs of inflammation.	Day 28: Alendronate induced bone exposure;	Day 28: Alendronate sustained a chronic inflammatory process	Day 28: Alendronate group presented a significant increase in areas with necrotic bone in interalveolar septum and alveolar crest
Khojasteh A et al., 2013^45^	n/a	n/a	n/a	n/a	Alendronate suppressed bone remodeling	Day 60: Alendronate increased empty osteocytic lacunae and bone sequestra	n/a	n/a	Day 60: Alendronate impaired epithelial coverage. Wounds were not healed completely.	Day 60: Alendronate induced mixed inflammation characterized by the combination of live and necrotic bone	Day 60: Alendronate induced osteonecrosis
Yamamoto-Silva FP et al., 2013^50^	Day 7: Alendronate induces atypical osteoclasts morphology; Day 21: Alendronate induced apoptosis in osteoclasts	Day 7,14 and 21: Alendronate decreases the number of osteoblasts	Day 7 and 21: Alendronate decreases woven bone (immature bone) while the formed immature bone filled less than 25% of their alveolar sockets.	Day 7, 14 and 21: Alendronate decreases bone formation. At day 7 (0%), 14 (≈10%) and 21 (≈20%) small amount of new bone formation was observed compared to the control group at days 7 (≈40%), 14 (≈60%) and 21 (≈60%).	Day 7,14 and 21: Alendronate inhibited crest resorption; Day 21: Alendronate suppressed alveolar bone remodeling	Day 14 and 21: Alendronate increased empty osteocytic lacunae	Day 7: Alendronate decreased new vessels formed in the apical region (endoglin, CD105)	Day 7: Alendronate group presented significant inflammatory infiltrate; Day 14: The alendronate group presented with chronic inflammatory infiltrate in the connective tissue.	Day 7: Alendronate induced no epithelial coverage. Day 14: Alendronate impaired epithelial coverage, which presented bacterial contamination.	Days 7 and 14: Alendronate increased inflammatory infiltrate	Day 14: Alendronate increased bacterial contamination and necrotic bone
Yamazaki T et al., 2013^51^	Day 14: Alendronate increased the number of nonfunctional osteoclasts, exhibiting lack of ruffled border situated away from the bone surface	Day 7: Alendronate decreased the number of osteoblasts	Days 7, 14 and 28: Alendronate increased granulation tissue	Day 7: The alendronate group showed small amount of new, immature bone of extraction. Day 14: newly formed bone in the socket has an irregular mesh and granulation tissue. The newly formed bone (≈60%) lacks the bone-like fiber bundle. Day 28: Alendronate decreased bone formation.	Day 7: Resorption of the interalveolar septum was detected.Day 14: Alendronate inhibits crest resorption. Day 14 and 28: Alendronate suppressed bone remodeling, and thickening the cavity wall	n/a	n/a	Day 14; Alendronate group showed that the newly formed bone lacks the bonelike fiber bundle	n/a	n/a	n/a
Tanoue R et al., 2015^49^	Day 10: Alendronate decreased the number of osteoclasts	n/a	n/a	Days 3,5,7,10 and 21: Bone formation in the alendronate group was not different compared to the control group in any of the periods evaluated.	Days 3,7 and 10: Alendronate increased the number of neutrophils and suppressed bone remodeling	Days 3,7 and 10: Alendronate increased the average percentage of the empty osteocytic lacunae	Days 3,5,7,10 and 21: blood vessel area was not significant compared to the control animals; Days 7 and 21: Alendronate decreased lymphatic vessel area	Day 21: Alendronate decreased collagen apposition (≈65%) compared to the control group (≈80%)	Day 10: epithelial coverage was not found in all the wounds in the alendronate and control groups; Day 21: Total epithelial coverage was observed in the alendronate and control groups (100%).	Days 5,7,10 and 21: Alendronate increased the number of neutrophils and sustained inflammation	n/a
Conte Neto N et al., 2016^41^	n/a	n/a	n/a	Day 28: Alendronate decreased bone formation (≈40%) compared to the control group (≈80%).	Day 28: Alendronate suppressed interalveolar septum bone remodeling, which was associated with bone necrosis and infection.	Day 28: Alendronate increased empty osteocytic lacunae	Day 28: Alendronate decreased vascularization	n/a	Day 28: Alendronate induced bone exposure with a marked impairment of alveolar socket re-epithelialization compared to the control group.	Inflammation in the alendronate group was not significantly different compared to the control group.	Day 28 Alendronate increased area of necrotic bone in the interadicular septum
Saulacic N et al., 2020^48^	Week 8: Alendronate increased the number of non-attached osteoclasts in the bone surface.	n/a	Week 8: Formation of woven bone from the original bone at the apical and lateral portion of the extraction socket	Week 8: Alendronate treatment increased vertical distance between the lingual and the buccal bone crest at root sites (1.48±1.37) compared to control group (1.28±0.28); and at extraction socket (2.16±0.74) and (0.76±0.05) in the alendronate and control group, respectively.	Week 8: Alendronate suppressed bone remodeling and did not prevent resorption of the buccal bone of the extraction socket. Percentage of mineralized bone was non-significant between alendronate and control (24.9±22.63; 35.68±32.61) as well as the total bone (39.9±34.9; 30.42±28.32) for alendronate and control	n/a	n/a	Week 8: Connective tissue was not significant different between alendronate (39.9±34.99) compared to control (30.42±28.32)	Alendronate did not impair epithelial coverage and the extraction socket was covered with oral mucosa lined with a keratinized epithelium.	Modest signs of inflammation at one week; no inflammation after 2 and 8 weeks	n/a
Isaias PHC et al., et al^52^	Some osteoclasts with signs of intracytoplasmic vacuolization and a few small bone sequestrations were observed in animals continuously treated with alendronate at doses of 5.0 and 7.5 mg/kg	n/a	The animals treated continuously with alendronate at the highest dose (7.5 mg/kg) showed significantly larger areas of fibrous tissue filling the alveoli of the left lower first molar	Animals in the alendronate group had areas of their extraction sites partially filled with bone, fibrous connective tissue, and mononuclear inflammatory infiltrate	Few small bone sequestrations were observed in animals continuously treated with alendronate at doses of 5.0 and 7.5 mg/kg	The bone tissues exhibited empty osteocyte lacunae in alendronate-treated animals (Fig. 5f–h), but to a lesser extent when compared to ZA group	n/a	n/a	Animals in the alendronate group had areas of their extraction sites partially filled with fibrous connective tissue	Alendronate increased mononuclear inflammatory infiltrate	Alendronate did not induce osteonecrosis at doses of 2.5, 5.0 and 7.5 mg/kg
Goncalves FC et al., 2022^42^	n/a	n/a	n/a	Day 30: Alendronate impaired socket healing with rats presenting extraoral fistulas, edema, and exposed bone	Day 30: Alendronate maintained bone volume; however, the bone was necrotic	Day 30: Alendronate increased the areas of empty osteocytic lacunae	n/a	Day 30: Alendronate did not impair fibroblast content	Day 30: Alendronate group presented with extraoral fistulas and exposed bone.	Day 30: Alendronate increased inflammatory infiltrate in the alveolar socket	Day 30: Alendronate increased the amount of necrotic bone (28.2±24.9%) compared to the control group (17.3±13%)
Goncalves FC et al., 2023^53^		n/a	n/a	Alendronate showed greater maintenance of mineralized tissues in the alveolar process after tooth extraction compared to the CTR.	Alendronate suppressed alveolar bone remodeling with great amount of necrotic bone	The interadicular and interdental septa of the extraction region were necrotic, characterized by the absence of osteocytes and empty gaps and a lack of bone resorption	n/a	n/a	In the alendronate group, the alveoli presented without epithelial tissue cover	Alendronate did no increase inflammatory infiltrate in the alveolar socket	The alendronate group presented more necrotic bone than the other groups (24.60% vs. 0.00% – CTR and ALN/IRL; 8.73% ALN/RL

Suppression of bone remodeling in animals receiving alendronate therapy was corroborated in most of the included studies.^10,11,38,41,43,45,46,48-53^ In all 10 studies that analyzed non-vital bone content,^10,39,41,42,45,46,49,50,52,53^ alendronate therapy showed the worst results, and in 13 out of 14 reports, alendronate reduced bone remodeling.^10,11,38,41-46,50-53^ In seven out of nine studies, alendronate hindered epithelial coverage.^11,41,45,47,51-53^ For instance, the study made by Aguirre et al.^10^ showed that in three out of nine alendronate-treated rats (33%), the interdental alveolar bone exceeded the superficial surface of the socket, which was occupied by inflammatory tissue and not covered by oral epithelium. In these cases, the interdental bone appeared to protrude into the oral cavity. These data were corroborated by a study made by Maahs et al.,^47^ which demonstrated that alendronate increased the loss of mucosal integrity by 72.7%. Previous studies have also shown that alendronate treatment impaired epithelial coverage with concomitant exposed bone to the oral environment.^11, 41, 42, 45^ In six out of 10 studies, alendronate therapy postponed inflammation resolution^11,45,50-53^ by increasing the number of neutrophils and the inflammatory infiltrate.^11,45,49,50^ All studies that analyzed osteoclasts unanimously affirmed that alendronate somehow impaired osteoclast activity and function or altered their morphology.^10,38,43,46,49-53^ For example, Altundal et al.^38^ showed that the number of osteoclasts in animals treated with alendronate decreased compared to control animals, which is in agreement with a study c by Hikita et al.^43^ Yamamoto-Silva et al.^50^ and Yamazaki et al.^51^ showed that alendronate therapy induces atypical osteoclast morphology and non-functional osteoclasts characterized by the lack of ruffled border and large distance from the bone surface. Recently, Isaias et al.^52^ have also demonstrated that alendronate therapy induced osteoclast formation with signs of intracytoplasmic vacuolization at the doses of 5.0 and 7.5 mg/kg.

Regarding empty osteocytic lacunae and bone sequestra, Aguirre et al.^10^ showed that animals treated with alendronate displayed a 2.5-fold increase in the percentage of empty osteocytic lacunae. Kim et al.^46^ also showed that alendronate significantly increased empty osteocytic lacunae (74.33 ± 10.50) compared to the control (41.67 ± 15.50), which was corroborated by other studies.^41,42 45 49,50^

In four out of six studies, alendronate hindered angiogenesis^11,38,41,50^ and inhibited lymph angiogenesis in one study.^49^ In three out of four studies, it reduced collagen apposition rates,^49-51^ and in four out of five studies, it diminished the number or functions of osteoblasts.^10,38,50,51^ Socket healing was also investigated by microcomputed tomography in six studies.^40,42,43,46,51,53^ Three of them showed lower bone density in the alveolar socket at 7^46, 51^ and 30 days^51^ of healing in the alendronate group. Hikita et al.^43^ showed that the proportion of newly formed bone (BV/TV) increased in the alendronate group, ranging from 16.90 ± 8.66 at 3 days after tooth extraction to 74.99 ± 4.02 after 14 days.

No meta-analysis was conducted, due to the lack of homogeneous results for the construction of summary measures.

## Discussion

The results of this systematic review indicate that alendronate monotherapy negatively affects the early phase of wound healing after tooth extraction in preclinical studies. Our findings suggest that alendronate administration restrains bone resorption, delaying the alveolar socket healing process .

The retrieved studies in this review used rats as the most frequent animal model to study socket healing after tooth extraction under alendronate administration. The advantages associated with this animal model include rapid bone turnover, convenient size, ease of housing house and care and, particularly, low purchasing and maitenance costs.^56^ On the other hand, some authors cited a few constraints related to the rat model for socket healing, such as limited alveolar bone content, high prevalence of root fractures during tooth extractions, dissimilar bone morphology compared to larger mammals with the absence of Haversian systems, and low rate of cortical bone remodeling. Furthermore, rats do not reach a true skeletal maturity due to their continuos growth throughout their lifespan.^57^

To better assess the therapeutic effects of antiosteoporotic drugs, the animal model should display a postmenopausal bone loss similar to that of humans. Bilateral ovariectomy is the most common techique for estrogen depletion, leading to bone loss and providing a useful model for the prevention and treatment of osteoporosis. Some authors feed the animals a low calcium and phosporous diet to optimize ovariectomy,^40, 58, 59^ while others combine glucocorticoid therapy with ovariectomy and alendronate.^60^ Unfortunately, ovariectomy was performed in only three reports^40, 44, 51^ and, accordingly, 16 out of 19 reports were not able to answer whether alendronate can reverse low bone density or prevent bone loss, presenting an osteoporosis-induced challenge.

Rats under 3 months of age do not reach the peak bone mass, so the assessement of bone loss can be misleading in such young animals. Considering the rapid growth of these animals, the lower bone mass could be due to impaired bone growth rather than to accelerated bone loss, as observed after menopause.^57^ In the 3-month-old mature rat model, bone growth slows down considerably, which allows for the simulation of menopausal experience. In this review, only three studies employed animals older than 3 months, which is considered an appropriate model for postmenopausal bone loss.^39,45,47^

Another important concern when exploring the effects of pharmaceutical drugs in animal models is the clinically equivalent dose. BPs are effective inhibitors of bone resorption, depending on the dosage.^2^ This reveals the researchers’ general tendency to increase doses in experimental trials in order to obtain positive results, increasing the clinically equivalent dose and, consequently, drug toxicity. To avoid misleading results, investigators should normalize the medication through an allometric method.^54^ The metabolic dose is considered the best method to achieve a more reliable equivalent dose, but there is no universally accepted means to do that. To compare effective doses, Marie^61^ states that we should use drug concentration in the circulating serum. In this review, only three studies^10,40,52^ performed an allometric test (body mass), and four studies^10,39,45,52^ reached the clinical dose of alendronate for osteoporosis treatment, 70 mg/week,^10^ assessed by the metabolic dose.

The methodological bias and dosage limitations do not allow drawing conclusions about the efficiency of a drug. So, this should be the starting point of an animal study that aims to test the efficancy of a drug. At the alendronate dose of 0.05 mg/kg/week by oral gavage,^47^ animals were about 300-fold underexposed and at a dose of 2.5 mg/kg/day given subcutaneously,^50^ animals were 93-fold overexposed. Can we extrapolate these results to humans? Doses used in these studies were far from relevant to the effective dose used in humans. One very important aspect to consider in normalizing a drug dose is the absorption concentration in the bloodstream. Alendronate given subcutaneously is nearly 100% absorbed, while in oral gavage, its absorption is about 1%.^2,62^ Maahs et al.^47^ did not reach the clinical dose conversion at the dose of 0.05 mg/kg administered weekly, by oral gavage, referencing Lehman et al.,^63^ who employed the same dose daily instead of weekly. Researchers should be aware to establish the clinically equivalent drug dosage by a correct allometric test and, whenever possible, to assess the serum levels.

Seventeen out of nineteen studies performed histologic assessements.^10,11,37-39,41-43,45-53^ Ten reports,^10,38,42,43,46,49-53^ which described osteoclast characteristcs, revealed some functional impairment with cell activity reduction caused by alendronate. Once adsorbed onto bone mineral surfaces, due to its high afinity for hydroxyapatite, BPs come in close extracellular contact with osteoclasts. During the bone resorption process, BPs dissociate from the bone surface, followed by intracellular intake into osteoclasts by fluid phase endocytosis.^64^ In the cytoplasm, alendronate blocks the formation of intermediates along the mevalonate biosynthesis pathway. Specifically, it inhibits farnesyl pyrophosphate synthase (FPPS), a key enzyme in the mevalonate pathway that generates isoprenoid lipids, farnesyl pyrophosphate (FPP), and geranylgeranyl diphosphate (GGPP), utilized in sterol synthesis and in the post-translational modification of small GTP-binding proteins, essential for osteoclast function. Inhibition of FPPS impairs the prenylation process, thus causing alterations in important osteoclast functions, including cytoskeletal arrangement, membrane ruffling, trafficking of intracellular vesicles, and apoptosis. ^2^

The findings of this review corroborate those obtained for the toxicity effects of alendronate on osteoclasts. Osteoclast morphology alterations were seen at the socket healing site after alendronate intake, including smaller^51^ and atypical^50^ cells with abnormal nuclei^43,46, 51^ and lack of a ruffled border.^51^ Reduction in osteoclast number,^38, 43, 49^ function,^10,38,43,46,49-51^ and resorbed lacunae on bone surface^10,38^ were also described. Additionally, the increased number of apoptotic osteoclasts was verified in the alendronate group.^50^ In fact, bone remodeling is substancially affected by osteoclast impairment. Twelve studies^10,11,38,41,43,45,46,49-53^ showed that bones in the alendronate group became more dynamic in terms of bone remodeling and bone resorption, compared with the controls. As a consequence, there is retention of the interseptal bone height followed by a higher volume,^10,41^ increased thickness of the buccal and lingual alveolar sockets,^38^ and a detectable clear boundary between the alveolar bone and new bone.^41,51^ It is difficult to separate bone remodeling and bone apposition rates. This is because suppressed osteoclasts may directly or indirectly influence bone formation. Most studies, 10 out of 11, that evaluated the effect of alendronate therapy in socket bone filling, found a delay in the healing process compared with controls.^10,11,38,41-43,49,50,52,53^ Alendronate also reduced the eroded surface of interalveolar septum by 90%.

that the histologic analysis of the alendronate group revealed that the newly formed bone lacked the bone-like fiber bundle after 14 days of healing, and it was also revealed that collagen content was reduced in alendronate specimens at 21 days after extraction, suggesting a compromised collagen production.^51^ The woven bone was quantified in four studies.^10,43,46,50^ Three of them^10,43,46^ detected less woven bone apposition rates at alveolar bone healing sites in alendronate-treated animals. Aguirre et al.^10^ showed that alendronate intake decreased 55% and 75% of woven bone volume compared to the control group, at moderate and high dosages, respectively. Taking into account post-extraction socket healing, according to Araújo et al.,^65^ these findings suggest that alendronate therapy delays the socket healing process, extending the inflammatory phase and postponing the proliferative phase. Four reports^11,45,49,50^ in this review corroborate the increased inflammatory response, posing a challenge to alendronate exposure in the early phase of socket healing. Impairment in vascular sprouts^10,11,41,50^ and lymphatic vessels^49^ may be the reasons for the delayed clearance and sterilization processes at the healing site, leading to a delayed tissue granulation production and its replacement by a provisional connective tissue matrix. Additionally, alendronate therapy also affected ephitelial coverage in seven reports,^11,41,45,47,50,52,53^ leading to a loss of mucosal integrity associated with bacterial infection. The toxicity effect of alendronate on the oral epithelium has been described,^5^ and this is one of the main reasons for ONJ development. Five studies out of seven^11, 41,42^ identified the presence of osteonecrosis at socket healing sites associated with alendronate after tooth extraction, and eight studies^10,37,41,42,45,46,50,52^ showed higher levels of empty osteocyte lacunae in the socket walls and interseptal bone, which is related to suppressed bone remodeling.^27^

The quality assessment of eligible studies in this systematic review was very hard to accomplish due to the lack of information provided by the authors. Many details regarding sequence generation, allocation concealment, and animals losses, are often unreported and were not recovered. To improve evidence-based animal experimentation, the authors should utilize a collaboration tool based on the Cochrane RoB tool^66^ for randomized clinical trials to enhance the efficiency of translating animal research results into clinical practice.

The results of this systematic review should be interpreted with caution mainly because the study design has some important limitations. For instance, this review included studies aimed at developing the BRONJ animal model utilizing the tooth extraction model and also articles dealing with management of bone remodeling after alendronate treatment. Therefore, of the wide variation in alendronate dosage and differences in the route of administration hinder the comparison of the effects of alendronate on the extraction socket. Besides, the heterogenous outcomes (animal age and strains, teeth extracted, measurements of outcomes, etc) of the included studies might also limit inferences about the effect of alendronate on socket healing. Therefore, more studies are needed to elucidate the potentially deleterious effect of alendronate on socket healing after tooth extraction in animal models.

## Conclusion

In summary, this systematic review identified that alendronate monotherapy negatively affects the early phase of wound healing after tooth extraction. It seems that alendronate affects the oral skeleton differently from other regions of the body. The reasons for that remain unclear and future research is needed to better understand the effects of alendronate on socket healing.
